# HOW DO MENTAL HEALTH TRANSITIONS SHAPE THE ROLE OF ADULT CHILDREN IN OLDER ADULTS’ SOCIAL NETWORKS?

**DOI:** 10.1093/geroni/igz038.227

**Published:** 2019-11-08

**Authors:** Laura Upenieks

**Affiliations:** University of Toronto, Toronto, Canada, Canada

## Abstract

This study considers the role of adult children in the core networks of older adults undergoing mental health change. Taking a multidimensional perspective of the network system, I consider (a) presence of child(ren), (b) contact with children network members, and (c) embeddedness of children within the network using longitudinal data from the United States. Parameters were estimated with generalized estimating equations from the pooled panel data. There was no evidence that mental health transitions lead to systematic forms of child reshuffling or increased contact with child ties. Children that remained in networks, however, showed increased contact with other members of the network when the parent underwent depression onset, but became less embedded when their parents had chronically high levels of depression. These patterns may have far-reaching consequences for older people and their children, which could include increased feelings of loneliness and social isolation for parent and child alike.

